# Genome-Wide Profiling of *WRKY* Genes Involved in Benzylisoquinoline Alkaloid Biosynthesis in California Poppy (*Eschscholzia californica*)

**DOI:** 10.3389/fpls.2021.699326

**Published:** 2021-06-17

**Authors:** Yasuyuki Yamada, Shohei Nishida, Nobukazu Shitan, Fumihiko Sato

**Affiliations:** ^1^Laboratory of Medicinal Cell Biology, Kobe Pharmaceutical University, Kobe, Japan; ^2^Department of Plant Gene and Totipotency, Division of Integrated Life Science, Graduate School of Biostudies, Kyoto University, Kyoto, Japan; ^3^Graduate School of Science, Osaka Prefecture University, Sakai, Japan

**Keywords:** benzylisoquinoline alkaloid, *Eschscholzia californica*, California poppy, *WRKY*, methyl jasmonate, RNA sequencing, tissue expression

## Abstract

Transcription factors of the WRKY family play pivotal roles in plant defense responses, including the biosynthesis of specialized metabolites. Based on the previous findings of WRKY proteins regulating benzylisoquinoline alkaloid (BIA) biosynthesis, such as CjWRKY1—a regulator of berberine biosynthesis in *Coptis japonica*—and PsWRKY1—a regulator of morphine biosynthesis in *Papaver somniferum*—we performed genome-wide characterization of the WRKY gene family in *Eschscholzia californica* (California poppy), which produces various BIAs. Fifty *WRKY* genes were identified by homology search and classified into three groups based on phylogenetic, gene structure, and conserved motif analyses. RNA sequencing showed that several *EcWRKY* genes transiently responded to methyl jasmonate, a known alkaloid inducer, and the expression patterns of these *EcWRKY* genes were rather similar to those of BIA biosynthetic enzyme genes. Furthermore, tissue expression profiling suggested the involvement of a few subgroup IIc EcWRKYs in the regulation of BIA biosynthesis. Transactivation analysis using luciferase reporter genes harboring the promoters of biosynthetic enzyme genes indicated little activity of subgroup IIc EcWRKYs, suggesting that the transcriptional network of BIA biosynthesis constitutes multiple members. Finally, we investigated the coexpression patterns of *EcWRKY*s with some transporter genes and discussed the diversified functions of *WRKY* genes based on a previous finding that CjWRKY1 overexpression in California poppy cells enhanced BIA secretion into the medium.

## Introduction

Being sessile, plants have evolved a wide array of defense mechanisms to protect themselves from diverse environmental stresses. WRKY transcription factors (TFs), one of the most important transcriptional regulators, play pivotal roles in plant development, senescence, and defense responses (Eulgem and Somssich, 2007; Rushton et al., 2010). The WRKY family proteins harbor at least one highly conserved WRKY domain composed of 60 amino acid residues, which includes the conserved N-terminal WRKYGQK sequence followed by a C-terminal zinc finger motif (Eulgem et al., 2000). The WRKY family can be divided into three groups (I–III). Group I proteins generally harbor two WRKY domains and a C2H2-type zinc finger motif. Group II proteins harbor a single WRKY domain and a C2H2-type zinc finger, and these can be further classified into five subgroups (IIa–IIe). Group III proteins also harbor a single WRKY domain and a C2HC-type zinc finger-like motif. The WRKY proteins modulate the expression of target genes by binding to the W-box DNA motif (C/TTGACC/T) in their promoter regions (Ulker and Somssich, 2004). The structures of several WRKY proteins indicate that the conserved WRKYGQK motif with a β-sheet structure binds to the major groove of the DNA strand of W-box sequence (Yamasaki et al., 2013). The RKYGQK residues are directly involved in DNA binding through extensive hydrophobic contacts with the methyl groups of thymine (Yamasaki et al., 2012).

In several species, WRKY family proteins regulate plant-specific (secondary) metabolism related to defense response against biotic and abiotic stresses (Yamada and Sato, 2013). For instance, GaWRKY1 regulates sesquiterpene biosynthesis in *Gossypium arboreum* (Xu et al., 2004). AaWRKT1 and GLANDULAR TRICHOME-SPECIFIC WRKY1 (AaGSW1) positively regulate antimalarial artemisinin biosynthesis in *Artemisia annua* (Ma et al., 2009; Chen et al., 2017). CrWRKY1 acts as an activator of monoterpenoid indole alkaloid biosynthesis via binding to the *tyrosine decarboxylase* (*TDC*) gene promoter in *Catharanthus roseus* (Suttipanta et al., 2011). The expression of these *WRKY* genes could be clearly induced by methyl jasmonate (MeJA)—a crucial phytohormone involved in plant defense and plant-specific metabolism—indicating that the WRKY TFs involved in the regulation of specialized metabolism in plants play important roles in the jasmonic acid (JA) signaling cascade.

Furthermore, the biosynthesis of benzylisoquinoline alkaloids (BIAs), which are pharmaceutically important and structurally divergent specialized chemicals (e.g., analgesics morphine and codeine are found in *Papaver somniferum*, and antimicrobial berberine in *Coptis japonica*), is also regulated by the WRKY TFs CjWRKY1 and PsWRKY (Kato et al., 2007; Mishra et al., 2013). In *C. japonica*, belonging to the Ranunculaceae family, CjWRKY1 specifically regulates the expression of berberine biosynthetic enzyme genes by binding to several W-boxes in their promoters (Kato et al., 2007; Yamada et al., 2016). In *P. somniferum*, belonging to the Papaveraceae family, PsWRKY plays an important role in wound-induced regulation of morphine biosynthesis (Mishra et al., 2013). Although the functions of both *WRKY* genes were induced by MeJA, they were classified into different groups of the WRKY family: CjWRKY1 in subgroup IIc and PsWRKY in group I.

*Eschscholzia californica* (California poppy), belonging to the Papaveraceae family, produces various BIAs, such as sanguinarine, chelerythrine, and escholtzine, which are different types of BIAs from berberine and morphine, while a common biosynthetic pathway from L-tyrosine to (*S*)-reticuline is shared. The main BIA found in *E. californica* is sanguinarine, which also produced by *P. somniferum* cultured cells. The biosynthetic pathways of sanguinarine and related BIAs have been intensively investigated at the molecular level (Supplementary Figure 1). Furthermore, the basic helix–loop–helix TFs EcbHLH1-1 and EcbHLH1-2 have been identified as the positive regulators of sanguinarine biosynthesis (Yamada et al., 2015). Recently, the draft genome sequence of California poppy was compiled and various gene families related to BIA biosynthesis in the genome of this plant were explored (Hori et al., 2018; Yamada et al., 2021). In fact, novel cytochrome P450 enzymes involved in macarpine biosynthesis and possible AP2/ERF TFs involved in the regulation of sanguinarine biosynthesis have been identified (Hori et al., 2018; Yamada et al., 2020).

Heterologous CjWRKY1 expression in *E. californica* cells strongly enhanced BIA biosynthesis, suggesting the involvement of WRKY protein(s) in the regulation of the BIA biosynthetic pathway in California poppy (Yamada et al., 2017). Although CjWRKY1 has been identified as a comprehensive regulator of almost all genes encoding berberine biosynthetic enzymes in *C. japonica*, ectopic CjWRKY1 expression in California poppy cells upregulated only a few BIA biosynthetic enzyme genes. Interestingly, CjWRKY1 overexpression in cultured California poppy cells enhanced BIA accumulation in the culture medium. Together, these findings suggest that the potential WRKY TF(s) involved in the regulation of the BIA biosynthetic pathway might be functionally diversified in BIA-producing plant species and gained additional functions associated with BIA production and accumulation in *E. californica*.

In this study, we investigated the WRKY family genes in the California poppy genome using gene annotation data and compared their expression profiles with those of *EcbHLH1* and some *EcAP2/ERF* genes involved in BIA biosynthesis. We classified the identified genes by phylogenetic analysis and performed gene structure and conserved motif analyses. The expression profiles of the *EcWRKY* genes in response to MeJA treatment were examined by RNA sequencing (RNA-Seq) and quantitative RT-PCR (qRT-PCR). Moreover, tissue-specific expression patterns of MeJA-responsive *EcWRKY* genes were investigated by qRT-PCR. We further searched for genes possibly involved in the efflux of BIAs and identified several transporter genes induced by MeJA, based on our previous finding of enhanced BIA secretion following CjWRKY1 overexpression in California poppy cells. The present characterization provides useful information on the physiological roles of *EcWRKY* genes and the transcriptional network of BIA biosynthesis in *E. caifornica*.

## Materials and Methods

### Identification of *WRKY* Genes From *E. californica*

First, 76 putative *WRKY* genes were isolated from the *E. californica* draft genome based on annotated gene information in the Eschscholzia Genome Database.^1^ Next, 20 genes that did not contain complete WRKY domain-encoding sequences were removed based on domain search using the SMART database,^2^ and six genes were removed because they harbored partial open reading frames or abnormal sequences, probably due to assembly errors. After sequence validation using the PhytoMetaSyn transcriptomic database^3^ (Xiao et al., 2013) and the NCBI database^4^ using BLAST (Supplementary Table 1), 50 *WRKY* genes were identified in the California poppy genome.

### Phylogenetic Analysis of *E. californica WRKY* TFs

The WRKY domain sequences of WRKY TFs from *Arabidopsis thaliana* and *E. californica* were obtained using the SMART database. Multiple sequence alignment was performed with ClustalW using BioEdit.^5^ An unrooted phylogenetic tree was created using MEGA 7.0.^6^ The neighbor-joining (NJ) method with the Jones–Thornton–Taylor (JTT) model and 1,000 bootstrap replications was used (Kumar et al., 2016).

### Genome Structure and Conserved Motif Analysis

The intron–exon organization of *E. californica WRKY* genes was visualized using the Gene Structure Display Server (GSDS)^7^ based on the predicted coding sequences and their corresponding genomic sequences. Conserved motifs of the EcWRKY proteins were predicted using MEME Suite (version 5.1.0)^8^ with the following parameters: maximum motif number of 15 and optimum motif width from ≥6 to ≤50 (Bailey et al., 2009). The topology of the phylogenetic tree was generated based on full-length WRKY protein sequences using MEGA 7.0.

### Plant Material

California poppy seedlings (“Hitoezaki”; Takii Seed Co., Ltd.) were grown and treated with 0.1% dimethyl sulfoxide (DMSO) as a control or 100 μM MeJA, as previously described (Yamada et al., 2020). The California poppy plants for tissue expression and metabolite analyses were grown in flowerpots for 5–6 months.

### RNA Sequencing and Expression Profiling Analyses

Total RNA extraction and sequencing were performed as described previously (Yamada et al., 2020) (Hokkaido Biosystem Science Co., Ltd., Hokkaido, Japan). The fragments per kilobase of exon model per million fragments mapped (FPKM) values were calculated using Cufflinks to evaluate gene expression levels. Hierarchical clustering was performed and heat maps were constructed based on log2-transformed fold change (FC) values compared to the mock control (0 h) using R.^9^

### qRT-PCR

Total RNA was extracted from six California poppy seedlings treated with 100 μM MeJA for 0, 0.5, 1, 2, 6, and 24 h, and tissues (leaf blade, petiole, root, flower bud, and flower) were obtained from nine plants using the RNeasy Plant Mini Kit (Qiagen, Hilden, Germany). Single-stranded cDNA was synthesized from to 500–1,000 ng of total RNA with the ReverTra Ace qPCR RT Master Mix using the gDNA Remover Kit (TOYOBO, Osaka, Japan). Real-time PCR was performed with specific primer pairs (Supplementary Table 2) using the THUNDERBIRD Next SYBR qPCR Mix (TOYOBO, Osaka, Japan) on the LightCycler 96 system (Roche, Basel, Switzerland). The PCR conditions were 95°C for 30 s, followed by 40 cycles of 95°C for 5 s and 60°C for 30 s. Gene expression levels were calculated using the 2^–ΔΔCt^ method to analyze MeJA response or generate a standard curve for tissue expression analysis. The relative expression levels were standardized to those of *actin* as the internal control.

### LUC Reporter Assay

The promoter:*LUC* constructs of the *Ec6OMT* and *EcCYP719A5* promoters have been constructed previously (Yamada et al., 2020). The full-length cDNAs of subgroup IIc *EcWRKY* genes were fused to the CaMV 35S promoter in the pBI221 vector, which was used as the effector construct. A dual-LUC reporter assay was then performed using *C. japonica* protoplasts, as previously described (Yamada and Sato, 2016).

### Metabolite Analysis

California poppy tissues were ground in liquid nitrogen and extracted overnight with 4 μL mg^–1^ fresh weight methanol containing 0.01 N HCl at room temperature (20°C). After filtration, the filtrate was prepared for metabolite analysis. Ultra-performance liquid chromatography (UPLC) equipped with QDa mass spectrometry was performed using the ACQUITY UPLC BEH C18 column (2.1 mm × 100 mm, 1.7 μm; Waters Corp.) operated at 40°C. Mobile phase A comprised an aqueous solution of 0.01% acetic acid, whereas mobile phase B comprised acetonitrile containing 0.01% acetic acid. Gradient elution was performed as follows: 0–1 min, 5% B; 1–13 min, 5-30% B; 13–17 min, 30–80% B; 17–18 min, 80–5% B; and 18–20 min, 5% B. The flow rate and injection volume were set at 0.3 mL min^–1^ and 2 μL, respectively. The QDa conditions were set as follows: cone voltage, 15 V; capillary voltage, 0.8 kV; and source temperature, 600°C. The predicted pavine-type BIAs were detected using total ion chromatography and mass spectrometry in the single-ion recording mode, and the fragmentation spectra (50 V cone voltage) were compared with previous data (Fabre et al., 2000).

## Results

### Identification and Classification of WRKY Family Members in the California Poppy Genome

To identify the WRKY TF-encoding genes in California poppy, we searched the *E. californica* draft genome database with gene annotation information using the sequence of a typical WRKY domain. After the removal of incomplete and redundant sequences, a total of 50 putative *WRKY* genes were identified in the California poppy draft genome, which were designated as *EcWRKY1* to *EcWRKY50* (Table 1). Of the 50 putative EcWRKY proteins, eight proteins possessed two WRKY domains, while the remaining proteins possessed only a single WRKY domain.

**TABLE 1:** Identified *WRKY* genes in the California poppy genome.

Gene name	Gene ID	Predicted ORF length	Subgroup
*EcWRKY1*	Eca_sc000058.1_g0310.1	1596	I
*EcWRKY2*	Eca_sc000993.1_g1210.1	1455	I
*EcWRKY3*	Eca_sc003413.1_g2610.1	1446	I
*EcWRKY4*	Eca_sc002150.1_g1640.1	1107	I
*EcWRKY5*	Eca_sc001139.1_g0510.1	2190	I
*EcWRKY6*	Eca_sc001139.1_g2480.1	828	III
*EcWRKY7*	Eca_sc000967.1_g1460.1	2118	I
*EcWRKY8*	Eca_sc016018.1_g0010.1	1893	I
*EcWRKY9*	Eca_sc000141.1_g0430.1	2364	I
*EcWRKY10*	Eca_sc194739.1_g0350.1	1362	I
*EcWRKY11*	Eca_sc194475.1_g0580.1	876	III
*EcWRKY12*	Eca_sc026098.1_g0870.1	933	III
*EcWRKY13*	Eca_sc194486.1_g1840.1	1635	IIb
*EcWRKY14*	Eca_sc000774.1_g0410.1	1005	III
*EcWRKY15*	Eca_sc002052.1_g0460.1	1062	I
*EcWRKY16*	Eca_sc001936.1_g0700.1	969	IIa
*EcWRKY17*	Eca_sc194540.1_g3890.1	1071	I
*EcWRKY18*	Eca_sc035472.1_g0020.1	915	IIe
*EcWRKY19*	Eca_sc014828.1_g0060.1	1017	IIe
*EcWRKY20*	Eca_sc194624.1_g0220.1	996	IIc
*EcWRKY21*	Eca_sc194624.1_g0620.1	1344	IIc
*EcWRKY22*	Eca_sc193975.1_g1270.1	1731	IIb
*EcWRKY23*	Eca_sc000153.1_g1770.1	816	IIc
*EcWRKY24*	Eca_sc000193.1_g1170.1	1293	IIb
*EcWRKY25*	Eca_sc188774.1_g0010.1	1836	IIb
*EcWRKY26*	Eca_sc001048.1_g0050.1	1383	IIe
*EcWRKY27*	Eca_sc014577.1_g1270.1	996	IId
*EcWRKY28*	Eca_sc194627.1_g0820.1	711	IIe
*EcWRKY29*	Eca_sc194627.1_g0450.1	1194	IIe
*EcWRKY30*	Eca_sc194718.1_g0150.1	525	IId
*EcWRKY31*	Eca_sc194541.1_g0680.1	777	IIc
*EcWRKY32*	Eca_sc194541.1_g0990.1	876	IIe
*EcWRKY33*	Eca_sc002191.1_g0270.1	1047	IIc
*EcWRKY34*	Eca_sc015821.1_g0220.1	954	IIc
*EcWRKY35*	Eca_sc003662.1_g0020.1	1125	IIe
*EcWRKY36*	Eca_sc001705.1_g0090.1	645	IIc
*EcWRKY37*	Eca_sc194693.1_g1320.1	1122	IIe
*EcWRKY38*	Eca_sc000585.1_g0210.1	1038	IIc
*EcWRKY39*	Eca_sc001875.1_g0400.1	1056	IIc
*EcWRKY40*	Eca_sc000725.1_g1020.1	978	IIc
*EcWRKY41*	Eca_sc000325.1_g1390.1	543	IIc
*EcWRKY42*	Eca_sc006961.1_g0400.1	687	IIc
*EcWRKY43*	Eca_sc013752.1_g0230.1	945	III
*EcWRKY44*	Eca_sc194480.1_g0450.1	990	III
*EcWRKY45*	Eca_sc194641.1_g0500.1	1035	IId
*EcWRKY46*	Eca_sc057080.1_g0140.1	744	IIc
*EcWRKY47*	Eca_sc001754.1_g0640.1	1041	IId
*EcWRKY48*	Eca_sc000537.1_g1450.1	738	IId
*EcWRKY49*	Eca_sc000399.1_g0520.1	1131	IId
*EcWRKY50*	Eca_sc000360.1_g0330.1	1035	III

**FIGURE 1:**
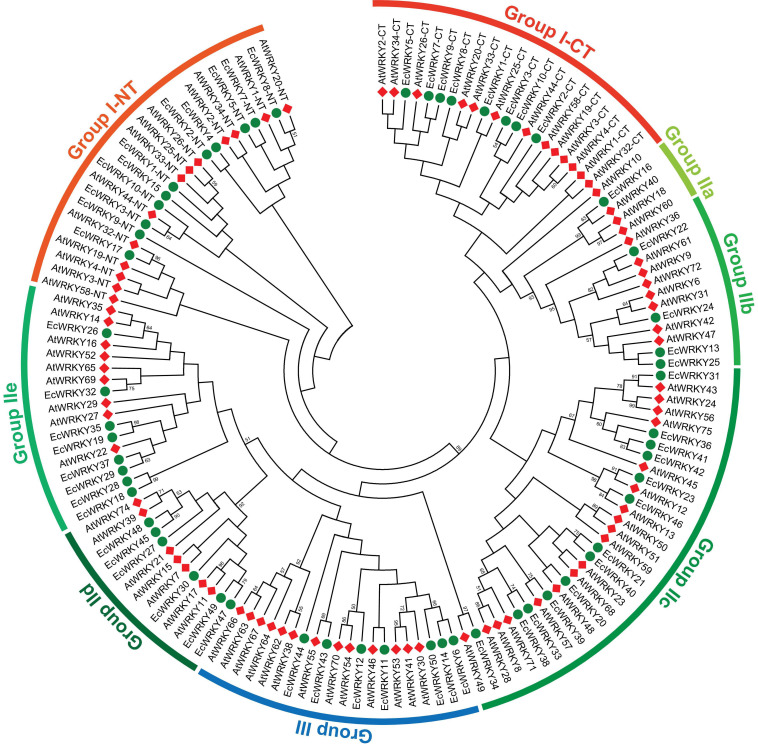
Phylogenetic tree of WRKY proteins in *Eschscholzia californica* and *Arabidopsis thaliana*. A neighbor-joining tree was constructed based on the amino acid sequences of the WRKY domains of 50 EcWRKY (green circle) and 72 AtWRKY (red diamond) proteins using MEGA 7.0. Bootstrap confidence values from 1,000 replicates are indicated at each branch.

To classify the 50 EcWRKY proteins, multiple sequence alignment using the WRKY domain of the 50 EcWRKY proteins and 72 AtWRKY proteins was performed, and an unrooted phylogenetic tree was constructed using the NJ method (Figure 1 and Supplementary Figure 2). Based on the classification of AtWRKY proteins and the phylogenetic tree, 11, 32, and 7 proteins were classified into groups I, II, and III, respectively (Table 1). Of the 32, respectively 1, 4, 13, 6, and 8 group II EcWRKY proteins were further divided into subgroups IIa, IIb, IIc, IId, and IIe. While majority of the group I WRKY proteins harbored two WRKY domains, three EcWRKY proteins in this group, namely EcWRKY4, EcWRKY15, and EcWRKY17, harbored only a single WRKY domain. Since the presence of group I WRKY proteins with a single WRKY domain has been reported in other plant species (Wei et al., 2012, 2016), these three EcWRKY proteins were classified as the group I WRKY proteins. California poppy has a similar number of WRKY groups to other plant species, with a similar number of genes in each group (Supplementary Table 3).

Homology search using CjWRKY1 and PsWRKY amino acid sequences as queries in the Eschscholzia Genome Database revealed high similarity of CjWRKY1 with three subgroup IIc WRKY proteins, namely EcWRKY36, EcWRKY41, and EcWRKY42. PsWRKY showed the highest similarity to group I EcWRKY1. A phylogenetic tree constructed using the WRKY domain sequences of the 50 EcWRKY proteins, CjWRKY1, and PsWRKY also showed the same result as the homology search (Supplementary Figure 3).

### Gene Structure and Conserved Motif Composition of the EcWRKY Family

**FIGURE 2:**
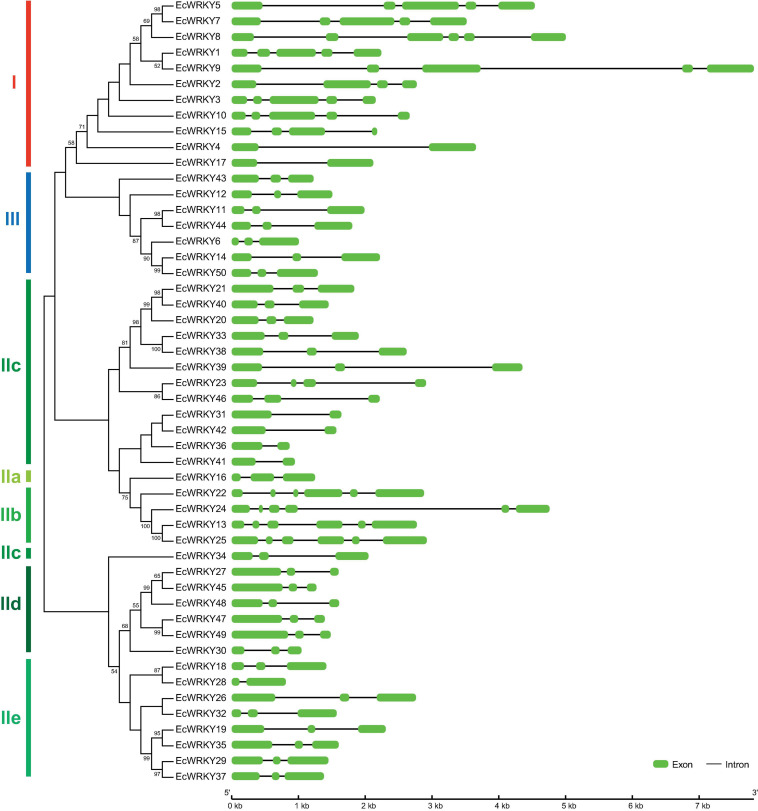
Structure of *EcWRKY* genes. A phylogenetic tree was constructed as shown in Figure 1, based on the amino acid sequences of the WRKY domains of 50 EcWRKY proteins. The intron–exon structure of the *EcWRKY* genes was visualized using GSDS. Green and black lines represent the exons and introns, respectively.

To compare the genomic DNA sequences of 50 *EcWRKY* genes, we determined their intron–exon structures (Figure 2). All *EcWRKY* genes had at least two exons, with 43 of the 50 *EcWRKY* genes having more than three exons. The distributions of introns and exons in the genomic sequences were relatively similar in each group. Most of the group I genes had four to five exons, except *EcWRKY4* and *EcWRKY17*, which had two exons. Furthermore, all subgroup IIb, IId, and III genes had six, three, and three exons, respectively. The phylogenetic tree indicated that subgroup IIc genes were divided into several clades. Four subgroup IIc genes (*EcWRKY31*, *EcWRKY36*, *EcWRKY41*, and *EcWRKY42*) in one clade had two exons, whereas the remaining nine genes in the other clades had three exons.

**FIGURE 3:**
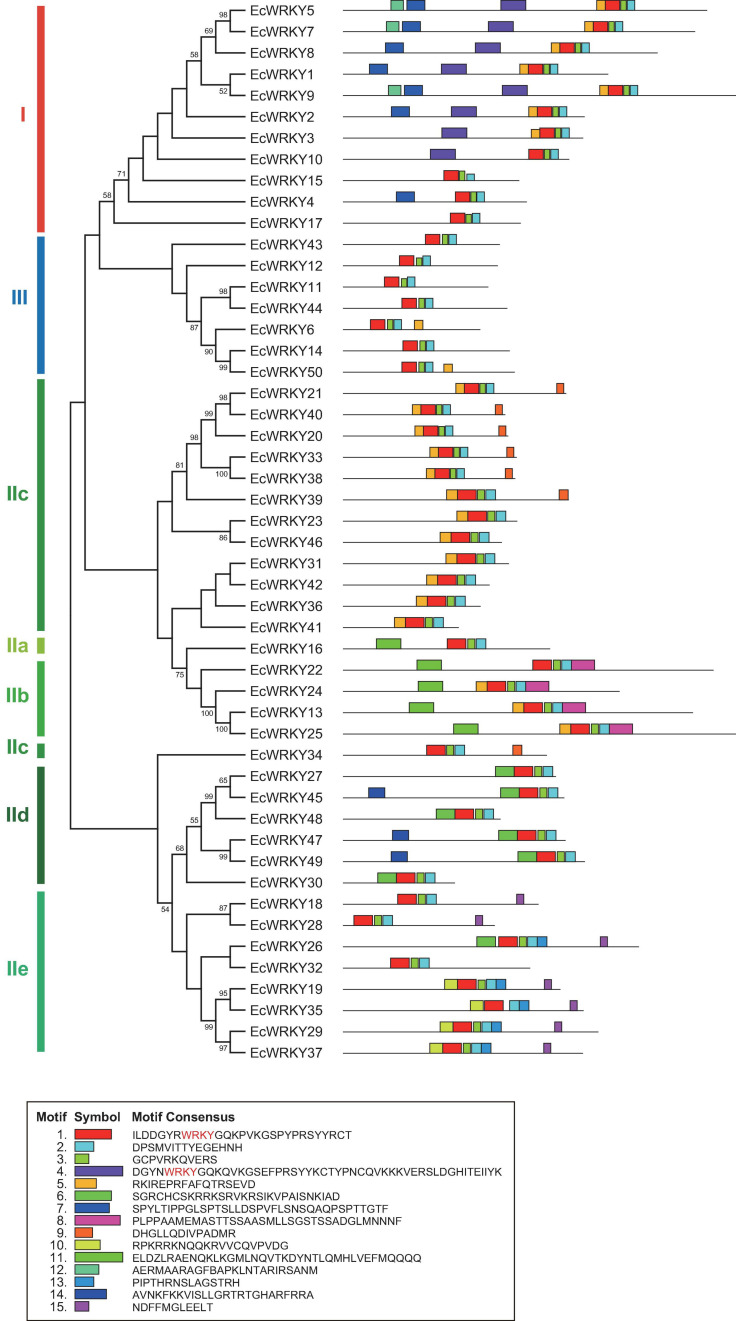
Conserved motifs of EcWRKY proteins. Fifteen motifs were identified using MEME and are indicated by colored rectangles. The height of the rectangles is proportional to the −log (*p*-value), truncated at the height of a motif with a *p*-value of 1e−10.

To examine the potential motifs of EcWRKY proteins in each family, we analyzed their conserved sequences using MEME Suite, a motif-based sequence analysis tool (Figure 3). Motifs 1, 2, and 3, which are components of the WRKY domain, were found in all EcWRKY proteins, while motif 4, which also contains the WRKYGQK core sequence, was only found in group I proteins, suggesting that motif 4 corresponds to the second WRKY domain. In addition to the WRKY domain, several conserved motifs were found in each EcWRKY family member. For example, motifs 7, 9, 11, and 15 were found only in (sub)group I, IIc, IIb, and IIe WRKY proteins, respectively. These conserved motifs might be important for the functional divergence of each protein group.

### MeJA-Induced Expression Profiling of *EcWRKY* Genes

**FIGURE 4:**
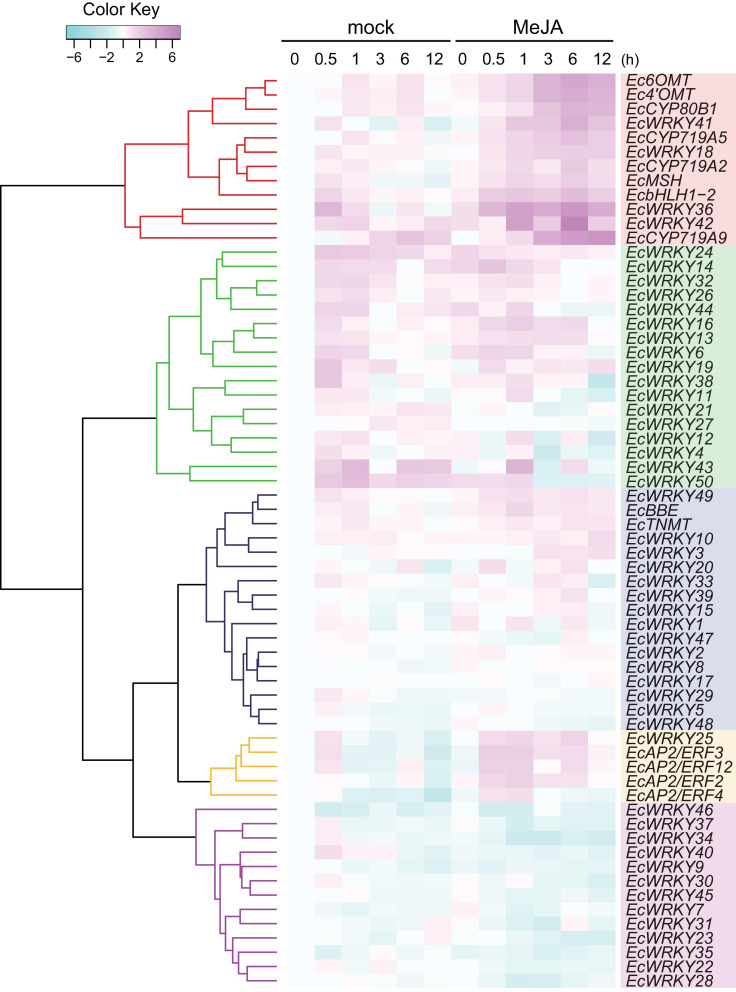
RNA sequencing-based expression profiles of *EcWRKY* genes following methyl jasmonate (MeJA) treatment. Heat maps showing the clustering of *EcWRKY* genes with *EcbHLH1-2*, *Ec6OMT*, *EcCYP80B1*, *Ec4’OMT*, *EcBBE*, *EcCYP719A2*, *EcCYP719A5*, *EcCYP719A9*, *EcTNMT*, *EcMSH*, *EcAP2/ERF2*, *EcAP2/ERF3*, *EcAP2/ERF4*, and *EcAP2/ERF12* were created using log2-based FPKM values in R. Within each row, low and high values are indicated in light blue and pink, respectively. The scale represents the signal intensity of FPKM values.

MeJA is an important phytohormone involved in defense response (Gundlach et al., 1992). Moreover, alkaloids play critical roles in protecting the plant body against pathogens and herbivores, and the expression of genes involved in the biosynthetic pathways of alkaloids, including BIAs, is strongly induced in response to MeJA (van der Fits and Memelink, 2000; Goossens et al., 2003; Ikezawa et al., 2007; Yamada et al., 2015). To investigate the MeJA responsiveness of *EcWRKY* genes, transcripts of California poppy seedlings treated with MeJA for 0, 0.5, 1, 3, 6, and 12 h were analyzed using RNA-Seq (Figure 4).

The expression of BIA biosynthetic enzyme genes (*Ec6OMT*, *Ec4’OMT*, *EcCYP80B1*, *EcCYP719A5*, *EcCYP719A2*, *EcMSH*, and *EcCYP719A9*) and TF genes (*EcbHLH1-2* and *EcAP2/ERFs*) were clearly induced in response to MeJA (Figure 4), as previously reported (Ikezawa et al., 2007, 2009; Yamada et al., 2015, 2020). The expression of *EcWRKY18*, *EcWRKY36*, *EcWRKY41*, and *EcWRKY42* was clearly increased following MeJA treatment. Hierarchical clustering indicated that these *EcWRKY* genes belong to the same clade as the MeJA-responsive BIA biosynthetic enzyme genes and *EcbHLH1-2*, whereas *EcAP2/ERF* genes, which showed earlier induction following MeJA treatment (Yamada et al., 2020), were placed in different clades, as discussed later. The expression profile of four *EcWRKY* genes was rather similar to that of BIA biosynthetic enzyme genes, which were strongly upregulated at 0.5–6 h (log2 FC > 1) and showed the greatest increase in expression after 6 h. In particular, *EcWRKY36* and *EcWRKY42* showed a more than 5-fold increase in expression after 6 h. Among the four EcWRKY TFs, EcWRKY36, EcWRKY41, and EcWRKY42 showed high similarity to CjWRKY1, as mentioned above (Supplementary Figure 3).

*EcBBE* and *EcTNMT* involved in the later stages of BIA biosynthesis were also upregulated by MeJA, although they belonged to a different clade from other biosynthetic enzyme genes. The expression of *EcWRKY3*, *EcWRKY10*, and *EcWRKY49* was weakly induced in response to MeJA, and these genes were placed in clade closely related to *EcBBE* and *EcTNMT* (Figure 4). *EcWRKY49* showed the highest expression after 1 h, whereas *EcWRKY3* and *EcWRKY10* showed the highest expression after 12 h, which suggested that EcWRKY49 might act as an early regulator in the JA signaling cascade to control other MeJA-responsive genes including other *EcWRKYs*. Both *EcWRKY3* and *EcWRKY10*, which encode group I proteins, showed relatively similar expression patterns in response to MeJA and their slower response to MeJA indicated that EcWRKY3 and EcWRKY10 might work further downstream of the JA signaling cascade.

Although *EcWRKY13*, *EcWRKY16*, and *EcWRKY25* genes did not show similar expression patterns to BIA biosynthetic enzyme genes in response to MeJA, their expression was upregulated (log2 FC > 1). *EcWRKY13* and *EcWRKY25* encode subgroup IIb proteins, whereas *EcWRKY16* encodes a subgroup IIa protein. Interestingly, the expression pattern of *EcWRKY25* was similar to that of the MeJA-responsive group IX *EcAP2/ERF* genes, which are the possible early regulators of BIA biosynthesis (Yamada et al., 2020).

**FIGURE 5:**
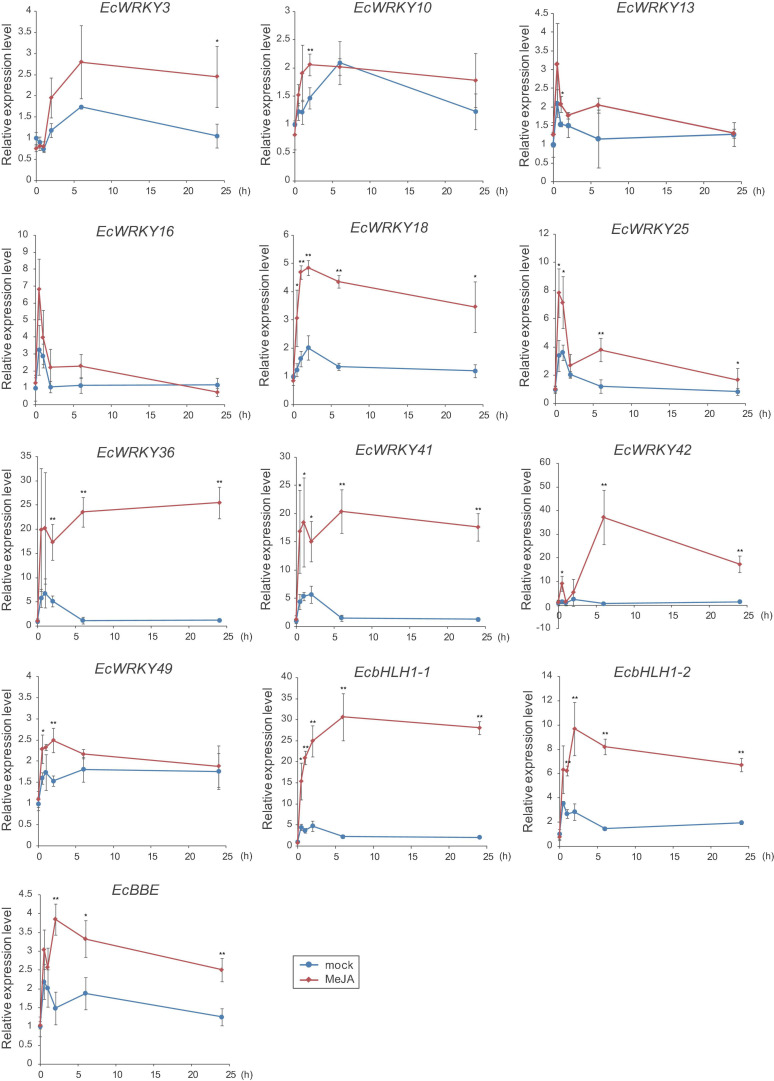
Expression levels of several *EcWRKY* genes in methyl jasmonate (MeJA)-treated seedlings. Expression levels of ten *EcWRKY genes*, *EcbHLH1-1*, *EcbHLH1-2*, and *EcBBE* were determined by qRT-PCR. The relative transcript levels represent the values standardized to those of the mock (0 h) samples set to 1. Error bars indicate the standard deviations calculated from three biological replicates. The asterisks denote significant differences according to Student’s *t*-test compared with the mocks: *P < 0.05; **P < 0.01.

To verify the expression profiles of *EcWRKY* genes that showed a clear increase in response to MeJA in RNA-Seq analysis, qRT-PCR was performed using cDNA derived from California poppy seedlings treated with MeJA for 0, 0.5, 1, 2, 6, and 24 h, with three biological replicates (Figure 5). As previously described (Yamada et al., 2015), two *EcbHLH1* and *EcBBE* genes were markedly upregulated in response to MeJA treatment, which is consistent with the results shown in Figure 4. The expression of *EcWRKY18*, *EcWRKY36*, *EcWRKY41*, and *EcWRKY42* was highly upregulated in response to MeJA treatment. The expression patterns of subgroup IIc *EcWRKY36*, *EcWRKY41*, and *EcWRKY42* were very similar. In contrast, MeJA did not strongly induce the expression of *EcWRKY3*, *EcWRKY10*, *EcWRKY13*, *EcWRKY16*, *EcWRKY25*, and *EcWRKY49* because of variation in gene expression in each seedling sample. Overall, these results indicate that *EcWRKY18*, *EcWRKY36*, *EcWRKY41*, and *EcWRKY42*, which showed a clear response to MeJA, are candidate *WRKY* genes involved in BIA biosynthesis.

### Expression Analysis of *EcWRKY* Genes in Different Tissues of California Poppy

**FIGURE 6:**
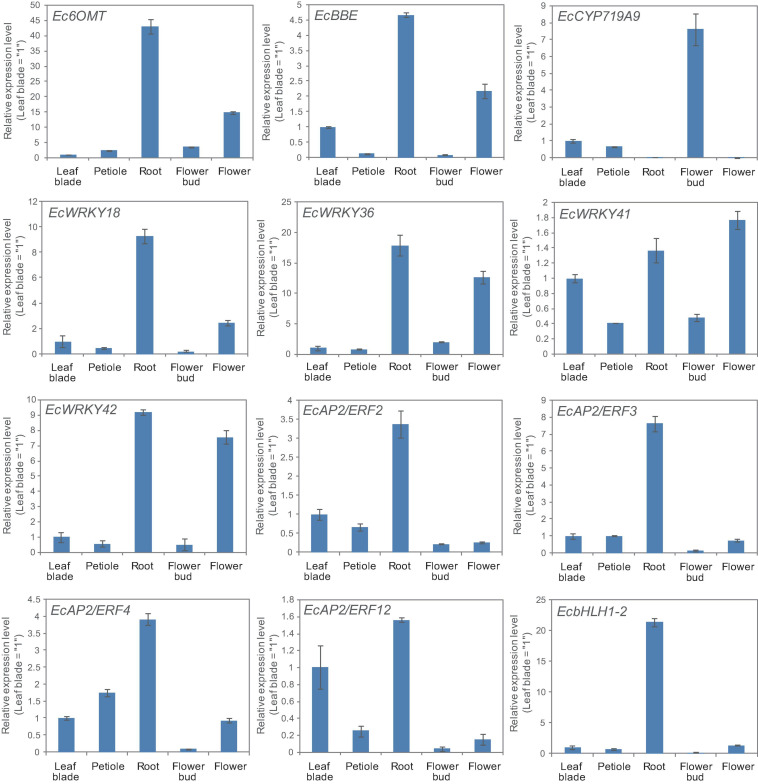
Tissue expression patterns of methyl jasmonate (MeJA)-responsive *EcWRKY* genes. The expression levels of four MeJA-responsive *EcWRKY* genes as well as those of *Ec6OMT*, *EcBBE*, *EcCYP719A9*, *EcbHLH1-2*, *EcAP2/ERF2*, *EcAP2/ERF3*, *EcAP2/ERF4*, and *EcAP2/ERF12* were determined by qRT-PCR using cDNA derived from nine California poppy plants. The relative transcript levels represent the values standardized to those of the leaf blade or petiole samples set to 1. Error bars indicate the standard deviations calculated from three technical replicates.

California poppy produces several types of BIA, which are accumulated in specific tissues. For instance, sanguinarine and chelerythrine are commonly accumulated in the root, whereas pavine-type BIAs, such as caryachine, californidine, and escholtzine, are only accumulate in aerial parts (Supplementary Figure 4). A previous study also revealed that genes involved in sanguinarine biosynthesis were highly expressed in roots (Ikezawa et al., 2007; Yamada et al., 2015). To further investigate the involvement of MeJA-responsive *EcWRKY* genes in the regulation of BIA biosynthesis, we examined the expression profiles of *EcWRKY18*, *EcWRKY36*, *EcWRKY41*, and *EcWRKY42* in different tissues, including leaf blades, petioles, roots, flower buds, and flowers (Figure 6) and compared them to the profiles of other TF genes involved in BIA biosynthesis, including *EcbHLH* and *EcERF*s. The expression profiles of *EcWRKY18* and *EcWRKY36* were highly similar to those of *Ec6OMT* and *EcBBE*, which encode sanguinarine biosynthetic enzymes; as such, these genes showed the highest expression in roots and relatively high expression in flowers. The expression profile of *EcWRKY42* was also similar to that of *EcWRKY18* and *EcWRKY36*, although it showed quite high expression in flowers as in roots. *EcbHLH1-2*, which is involved in sanguinarine biosynthesis, was highly and exclusively expressed in roots, as reported previously (Yamada et al., 2015). Meanwhile, group IX *EcAP2/ERF* genes were relatively highly expressed in leaves and roots, and these TF genes showed lower expression levels in flowers than *EcWRKY* genes. *EcCYP719A9*, encoding a possible enzyme involved in pavine-type BIA biosynthesis (Ikezawa et al., 2009), was highly expressed in aerial parts, particularly flower buds; however, the expression profile of any *EcWRKY* genes was not similar to that of *EcCYP719A9*. These results indicate that EcWRKY18 and subgroup IIc EcWRKYs, namely EcWRKY36, EcWRKY41, and EcWRKY42, are involved in the regulation of benzophenanthridine-type BIA biosynthesis.

### Role of Subgroup IIc EcWRKY Proteins in BIA Biosynthesis

Since EcWRKY36, EcWRKY41, and EcWRKY42 are putative CjWRKY1 homologs in the California poppy, we focused on these proteins and examined their transcriptional activity using a transient LUC reporter assay. While CjWRKY1 showed clear transcriptional activity in *C. japonica* cells (Kato et al., 2007; Yamada et al., 2016), EcWRKY36, EcWRKY41, and EcWRKY42 showed little induction of LUC activity derived from the *Ec6OMT* and *EcCYP719A5* gene promoter:*LUC* constructs (Supplementary Figure 5). These results are consistent with our previous findings that the expression of many biosynthetic enzyme genes, including *Ec6OMT* and *EcCYP719A5*, was not significantly upregulated in CjWRKY1-overexpressing California poppy cells. These results also suggest that the regulatory role of WRKY TFs in BIA biosynthesis might be diversified between *C. japonica* and *E. californica*, and EcWRKY proteins serve different functions in the BIA biosynthetic pathway.

### Coexpression Analysis of MeJA-Responsive *EcWRKY* Genes With Transporter-Encoding Genes

**FIGURE 7:**
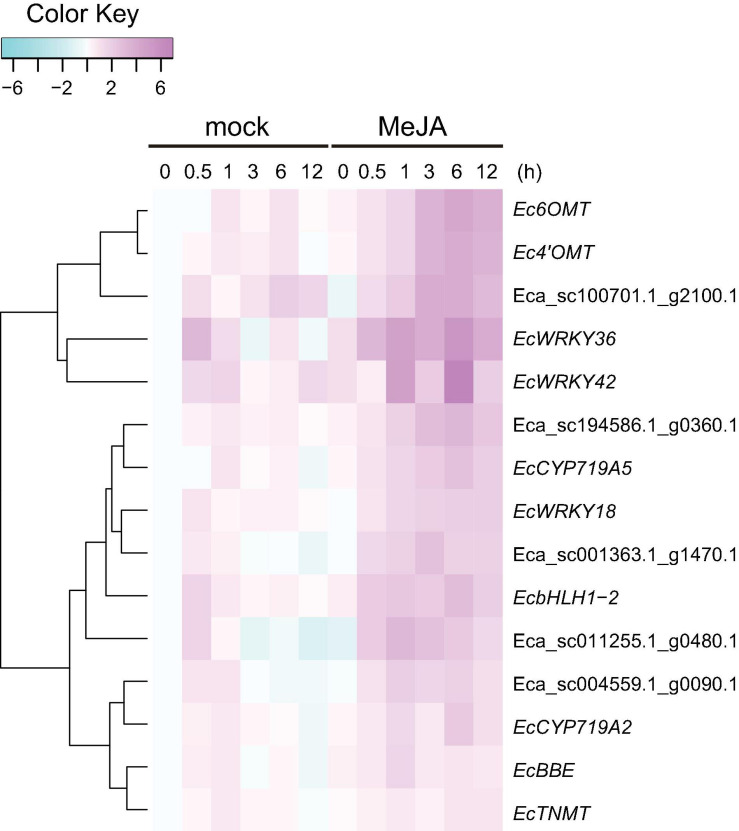
Expression profiles of putative transporter genes and *EcWRKY* genes following methyl jasmonate (MeJA) treatment. Heat maps showing the clustering of three ABCB transporter genes (Eca_sc001363.1_g1470.1, Eca_sc011255.1_g0480.1, and Eca_sc100701.1_g2100.1) and two MATE transporter genes (Eca_sc004559.1_g0090.1 and Eca_sc194586.1_g0360.1) with three *EcWRKY* genes (*EcWRKY18*, *EcWRKY36*, and *EcWRKY42*), *EcbHLH1-2*, *Ec6OMT*, *Ec4’OMT*, *EcBBE*, *EcCYP719A2*, *EcCYP719A5*, and *EcTNMT* were created using log2-based FPKM values. The scale represents the signal intensities of the FPKM values.

Our previous work revealed that heterologous expression of CjWRKY1 in California poppy cells increased BIA secretion into the culture medium (Yamada et al., 2017). This result suggests that the WRKY TFs modulate the expression of genes encoding transporter proteins involved in the efflux of alkaloids. To investigate the association between EcWRKY proteins involved in the regulation of genes encoding transporter proteins involved in the efflux of California poppy alkaloids, including BIAs, we explored transporter-encoding genes that showed similar expression patterns to *EcWRKY18*, *EcWRKY36*, and *EcWRKY42* in response to MeJA. We screened 46 transporter-encoding genes that were upregulated (log2 FC > 1) following MeJA treatment for 1–12 h (Supplementary Table 4). These candidates included two genes encoding multidrug and toxic compound extrusion (MATE) transporters and three genes encoding B-type ATP-binding cassette (ABC) transporters (Table 2), have a possibility to be involved in the translocation of alkaloids, such as berberine in *C. japonica* and nicotine in *Nicotiana tabacum* (Shitan et al., 2003, 2013, 2014; Morita et al., 2009; Shoji et al., 2009; Takanashi et al., 2017). Hierarchical clustering analysis revealed that the expression patterns of Eca_sc001363.1_g1470.1 and Eca_sc100701.1_g2100.1, which are putative B-type ABC transporter genes in response to MeJA, were relatively similar to those of *EcWRKY18* and *EcWRKY36* and *EcWRKY42*, respectively (Figure 7). Therefore, these ABCB transporter genes might be involved in the transport of BIAs and regulated by MeJA-responsive EcWRKY transcription factors.

**TABLE 2:** MeJA-responsive genes encoding ABC and MATE transporters.

Gene ID	Annotation	ORF length
**Eca_sc001363.1_g1470.1**	Nr = XP_010255510.1 PREDICTED: ABC transporter B family member 15-like [*Nelumbo nucifera*] Araport = AT3G28345.1 | ABC transporter family protein | Chr3:10593921-10598775 REVERSE LENGTH = 1240 | 201606	3,858
**Eca_sc004559.1_g0090.1**	Nr = XP_008796381.1 PREDICTED: protein DETOXIFICATION 33-like [*Phoenix dactylifera*] Araport = AT1G47530.1 | MATE efflux family protein | Chr1:17451724-17454110 FORWARD LENGTH = 484 | 201606	1,452
**Eca_sc011255.1_g0480.1**	Nr = XP_010271025.1 PREDICTED: ABC transporter B family member 11-like isoform X1 [*Nelumbo nucifera*] Araport = AT1G02520.3 | P-glycoprotein 11 | Chr1:524134-528745 FORWARD LENGTH = 1278 | 201606	3873
**Eca_sc100701.1_g2100.1**	Nr = XP_002279471.2 PREDICTED: ABC transporter B family member 13-like [*Vitis vinifera*] Araport = AT1G27940.2 | P-glycoprotein 13 | Chr1:9733597-9737211 REVERSE LENGTH = 1031 | 201606	2,685
**Eca_sc194586.1_g0360.1**	Nr = XP_010260247.1 PREDICTED: MATE efflux family protein LAL5-like [*Nelumbo nucifera*] Araport = AT3G23560.1 | MATE efflux family protein | Chr3:8454361-8456588 REVERSE LENGTH = 477 | 201606	1,440

## Discussion

Genes of the WRKY superfamily, which is one of the largest groups of TFs involved in plant development and response to various stresses, have been identified in various plants (Rushton et al., 2010). Recent advances in whole-genome sequencing technologies have enabled us to perform genome-wide analysis of *WRKY* genes in many plant species. To date, 74 *WRKY* genes have been identified in *A. thaliana* (Ulker and Somssich, 2004), 52 in *C. roseus* (Schluttenhofer et al., 2014), 54 in *Ananas comosus* (Xie et al., 2018), 55 in *Cucumis sativus* (Ling et al., 2011), 85 in *Manihot esculenta* (Wei et al., 2016), 70 in *Aquilaria sinensis* (Xu et al., 2020), and 120 in *Gossypium raimondii* (Cai et al., 2014). However, our study is the first report on the genome-wide identification of WRKY TFs from *E. californica*, a BIA-producing plant of the Papaveraceae family. We identified 50 WRKY members in the California poppy draft genome (Table 1). The different number of *WRKY* genes among plant species may be implicated in differences in the size of the genome and functional diversification of WRKY family proteins during evolution. Based on phylogenetic analysis (Figure 1), the 50 EcWRKY proteins were classified into 11 group I proteins; 32 group II proteins, further divided into 1, 4, 13, 6, and 8 proteins in subgroup IIa, IIb, IIc, IId, and IIe, respectively; and 7 group III proteins. The distribution of each group of WRKY proteins in *E. californica* was quite similar to that in other plant species, although there were lower subgroup IIa and IIb proteins in California poppy than those in other species (Supplementary Table 3). Since *E. californica* is a basal eudicot of the Papaveraceae family, this difference likely reflects the evolutionary history of land plant subgroup IIa and IIb genes, which are considered to have evolved from group I genes due to deletion of the domain structure (Wang et al., 2014).

Gene structure and conserved motif analyses indicated that each protein group shared a similar number of introns and similar motifs (Figures 2, 3). All *EcWRKY* genes possessed more than one intron, which is consistent with reports in other plant species (Wei et al., 2016; Xie et al., 2018; Xu et al., 2020). These results suggest that gene duplication and structural diversification of *WRKY* genes may have occurred at the early stages of evolution. Furthermore, the similar motif compositions of each WRKY protein group indicate the potential functional similarity among them, as three subgroup IIc *EcWRKY* genes, namely *EcWRKY36*, *EcWRKY41*, and *EcWRKY42*, showed a marked response to MeJA.

**FIGURE 8:**
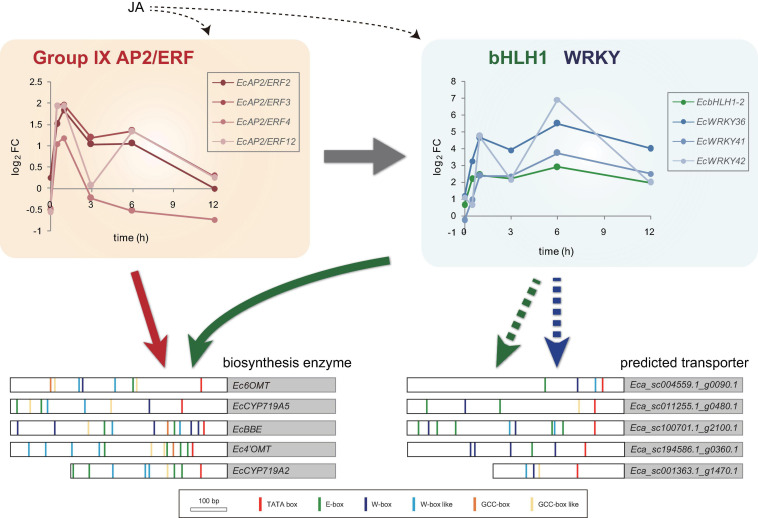
Predicted model of gene expression mechanism in benzylisoquinoline alkaloid biosynthesis via jasmonic acid (JA)-responsive transcription factors. Temporal expression patterns of group IX *EcAP2/ERF*, *EcbHLH1-2*, and *EcWRKY* genes are shown with log2 FC values based on the RNA-Seq data. Large white and gray boxes indicate the partial promoter and coding sequence, respectively. Small boxes with several colors represent the putative *cis*-acting elements. Based on the previous results, EcAP2/ERF and EcbHLH1 show transactivation of the promoters of biosynthetic enzyme genes (indicated by red and green arrows, respectively). Since the promoters of the predicted transporter genes harbor many putative E-box and W-box elements, EcbHLH and EcWRKY transcription factors may regulate them rather than EcAP2/ERF proteins (indicated by green and blue dotted arrows, respectively).

Jasmonic acid signaling is a critical axis in defense response, including the biosynthesis of specialized metabolites, which act as chemical defense compounds against herbivores and pathogens. Alkaloid production is modulated by JA signaling, and many JA-responsive TFs, which play vital roles in the regulation of genes involved in the JA signaling cascade, have been identified and characterized (Yamada and Sato, 2013). Hence, JA-responsive *WRKY* genes in *E. californica* may regulate the expression of genes involved in the BIA biosynthetic pathway. The results of RNA-Seq and qRT-PCR revealed that four *EcWRKY* genes, namely *EcWRKY18*, *EcWRKY36*, *EcWRKY41*, and *EcWRKY42*, were upregulated following MeJA treatment (Figures 4, 5). The response pattern of *EcWRKY* genes was relatively similar to that of BIA biosynthetic enzyme genes, including group IX *EcAP2/ERF* and *EcbHLH1-2*, which showed a rapid MeJA response (Yamada et al., 2020). These results indicate that EcWRKY TFs may function downstream of group IX EcAP2/ERF and EcbHLH1-2 TFs in the JA signaling cascade (Figure 8). To investigate the detailed transcriptional network of BIA biosynthesis in California poppy, further functional characterization of bHLH, AP2/ERF, and WRKY TFs using stable transformants is warranted. Furthermore, EcWRKY36, EcWRKY41, and EcWRKY42, which are potential CjWRKY1 homologs, showed little transcriptional activity in transient LUC assay using the *Ec6OMT* and *EcCYP719A5* gene promoters (Supplementary Figure 5), which is consistent with our previous results of CjWRKY1 overexpression in Californian poppy cells (Yamada et al., 2017). Therefore, the function of WRKY proteins involved in BIA biosynthesis may have diversified during evolution in *E. californica* and *C. japonica*, and EcWRKY proteins may serve additional functions contributing to BIA production. Additionally, post-transcriptional regulation might be involved in BIA biosynthesis such as protein phosphorylation and degradation (Yamada and Sato, 2016).

Group I WRKY proteins are involved in the regulation of BIA biosynthesis. For instance, PsWRKY has been identified as a potent transcriptional activator of BIA biosynthetic genes in *P. somniferum* (Mishra et al., 2013). Moreover, Apuya et al. (2008) reported that AtWRKY1 overexpression in *P. somniferum* and *E. californica* cultured cells enhanced BIA accumulation. In contrast, our RNA-Seq and qRT-PCR data revealed that the expression of group I *EcWRKY* genes, including *EcWRKY1*, the closest homologous gene of *PsWRKY*, was not or weakly induced in response to MeJA (Figures 4, 5). These results also suggest the functional diversification of group I WRKY proteins in the Papaveraceae family during evolution. However, whether *PsWRKY* modulates the expression of enzymes involved in sanguinarine or morphine biosynthetic pathways in *P. somniferum* remains unclear. Therefore, detailed functional characterization of group I WRKY proteins in BIA-producing plants is essential.

Our previous study revealed that CjWRKY1 overexpression in California poppy cultured cells enhanced BIA accumulation in culture medium (Yamada et al., 2017), suggesting that WRKY proteins regulate the expression of genes encoding potential transporters of BIAs in this plant. Several TFs regulating genes encoding transport proteins of specialized metabolites have been identified. For instance, *A. thaliana* MYB TFs regulate the expression of genes involved in the transport of proanthocyanidins (Sharma and Dixon, 2005), and grapevine MYB and WRKY TFs synergistically regulate the expression of genes involved in flavonoid accumulation (Amato et al., 2019). During alkaloid biosynthesis, the expression of genes encoding MATE transporters is regulated by bHLH and AP2/ERF TFs, which also control the expression of biosynthetic enzyme genes (Shoji et al., 2010; Takanashi et al., 2017). The compartmentalization of cytotoxic alkaloids in specific organs or organelles via transporters is important for protection against insects and herbivores, and the regulation of expression of such transporters is important; however, little is known regarding transporters involved in the compartmentalization of BIAs in *E. californica* cells. In this light, we investigated the coexpression patterns of *WRKY* and transporter genes in MeJA-treated *E. californica* seedlings. Three ABCB transporter and two MATE transporter genes that showed a clear MeJA response were coexpressed with *EcWRKY18*, *EcWRKY36*, and *EcWRKY42*. Interestingly, search for putative *cis*-elements using the New PLACE database^10^ in the promoter regions of MeJA-responsive transporter genes and biosynthetic enzyme genes (Higo et al., 1999) revealed that there were few GCC-box-like *cis*-elements, which are target sequences of group IX AP2/ERF TFs, in these transporter genes and at least one GCC-box or GCC-box-like nucleotide sequence was present in genes encoding biosynthetic enzymes (Figure 8). To reveal the direct interaction of these putative *cis*-elements with AP2/ERF, WRKY, and bHLH TFs, additional analyses are required in future studies. Furthermore, the predicted transporter-encoding genes that were highly upregulated by MeJA included many genes encoding nitrate transporter 1/peptide transporter family (NPF) proteins and purine permeases (Supplementary Table 4). CrNPF2.9 involved in the transport of strictosidine from vacuole to cytosol and BIA uptake purine permeases have recently been isolated from *C. roseus* and opium poppy, respectively (Payne et al., 2017; Dastmalchi et al., 2019). Therefore, MeJA-responsive NPF transporters and purine permeases might be involved in the translocation of BIAs in *E. californica*.

In conclusion, our genome-wide analysis and expression profiling of the WRKY family genes in *E. californica* would be useful for understanding the regulatory mechanisms underlying of BIA biosynthesis, accumulation, and translocation. Especially, different EcWRKY proteins might regulate the spatiotemporal expression patterns of genes related to BIA biosynthesis. Further characterization of EcWRKY TFs and transporters is required to elucidate regulatory mechanisms of BIA production and accumulation in California poppy. This information will contribute to the development of metabolic and transport engineering approaches for the efficient production of valuable alkaloids.

## Data Availability Statement

The datasets presented in this study can be found in online repositories. The names of the repository/repositories and accession number(s) can be found below: https://www.ddbj.nig.ac.jp/, BEHA01000001–BEHA01053253.

## Author Contributions

YY and FS conceived and designed the study and wrote the manuscript. YY and SN analyzed the genomic and transcriptomic data and performed the experiments. NS and FS supervised the project and discussed the results. All authors reviewed the manuscript.

## Conflict of Interest

The authors declare that the research was conducted in the absence of any commercial or financial relationships that could be construed as a potential conflict of interest.

## Footnotes

1 http://eschscholzia.kazusa.or.jp

2 http://smart.embl-heidelberg.de/

3 https://bioinformatics.tugraz.at/phytometasyn/

4 https://www.ncbi.nlm.nih.gov/

5 https://bioedit.software.informer.com/

6 http://www.megasoftware.net/

7 http://gsds.cbi.pku.edu.cn/

8 http://meme-suite.org/

9 https://www.r-project.org/

10 https://www.dna.affrc.go.jp/PLACE/?action=newplace
