# Supplementary material for: Genome-Wide Profiling of WRKY Genes Involved in Benzylisoquinoline Alkaloid Biosynthesis in California Poppy (Eschscholzia californica)
Source: Front Plant Sci. 2021 Jun 17;12:699326. doi: 10.3389/fpls.2021.699326 (PMC8248504; doi:10.3389/fpls.2021.699326)
Supplement: Supplementary file 1 [file Data_Sheet_1.PDF]

Supplementary Material

Supplementary Figures

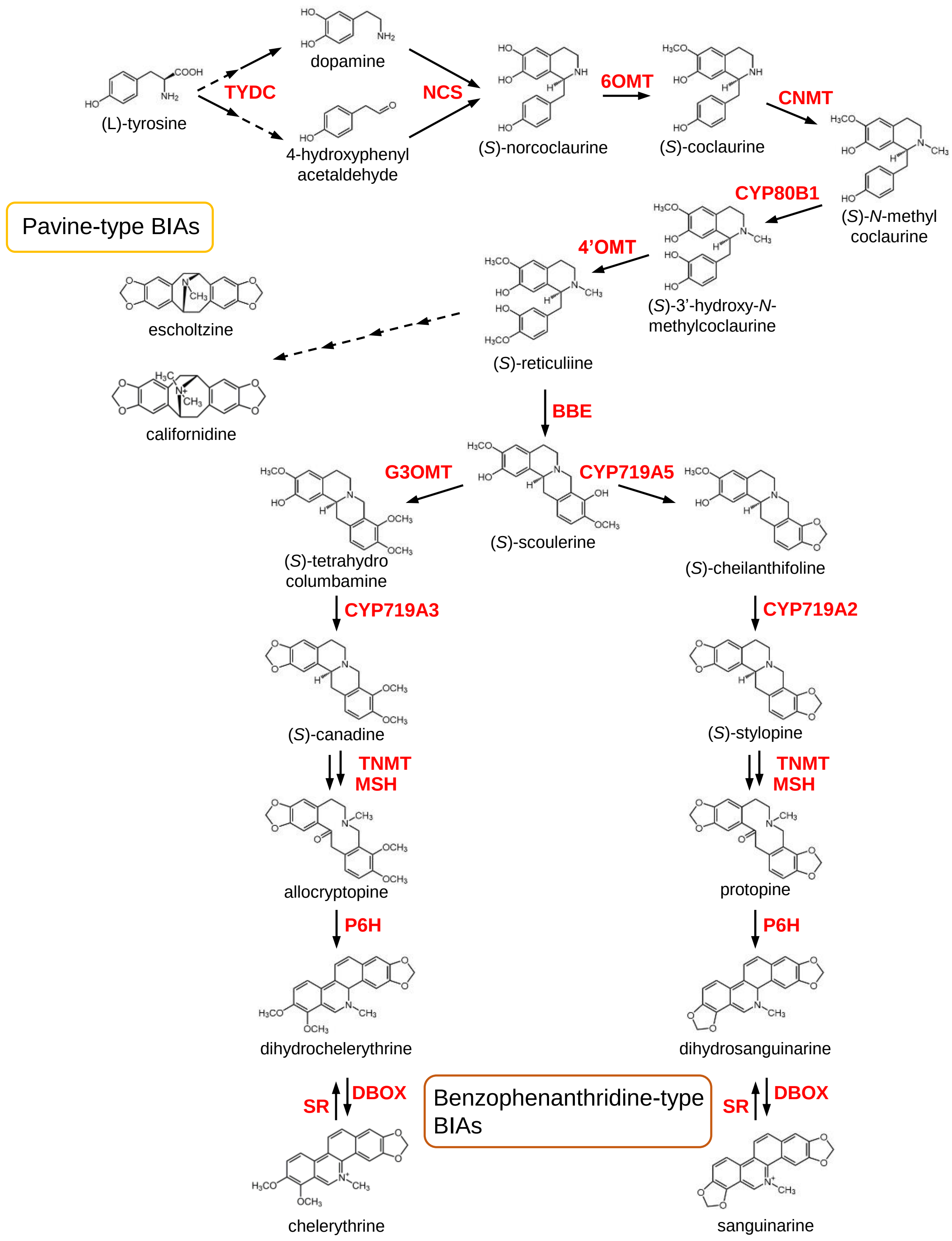

Supplementary Figure 1. Benzylisoquinoline alkaloid biosynthetic pathway in *Eschscholzia caifornica*. The broken lines indicate novel biosynthetic enzyme genes. TYDC, tyrosine/DOPA decarboxylase; NCS, (*S*)-norcoclaurine synthase; 6OMT, (*S*)-norcoclaurine 6-*O*-methyltransferase; CNMT, (*S*)-coclaurine-*N*-methyltransferase; CYP80B1, (*S*)-*N*-methylcoclaurine 3'-hydroxylase; 4'OMT, (*S*)-3'-hydroxy-*N*-methylcoclaurine 4'-*O*-methyltransferase; 7OMT, (*S*)-reticuline 7-*O*-methyltransferase; BBE, berberine bridge enzyme; G3OMT, (*S*)-scoulerine 9-*O*-methyltransferase; CYP719A2, (*S*)-stylopine synthase; CYP719A3, (*S*)-canadine/stylopine synthase; CYP719A5, (*S*)-cheilanthifoline synthase; TNMT, (*S*)-tetrahydroprotoberberine *N*-methyltransferase; MSH, (*S*)-*N*-methylstylopine 14-hydroxylase; P6H, protopine 6-hydroxylase; DBOX, dihydrobenzophenanthridine alkaloid oxidase; SR, sanguinarine reductase.

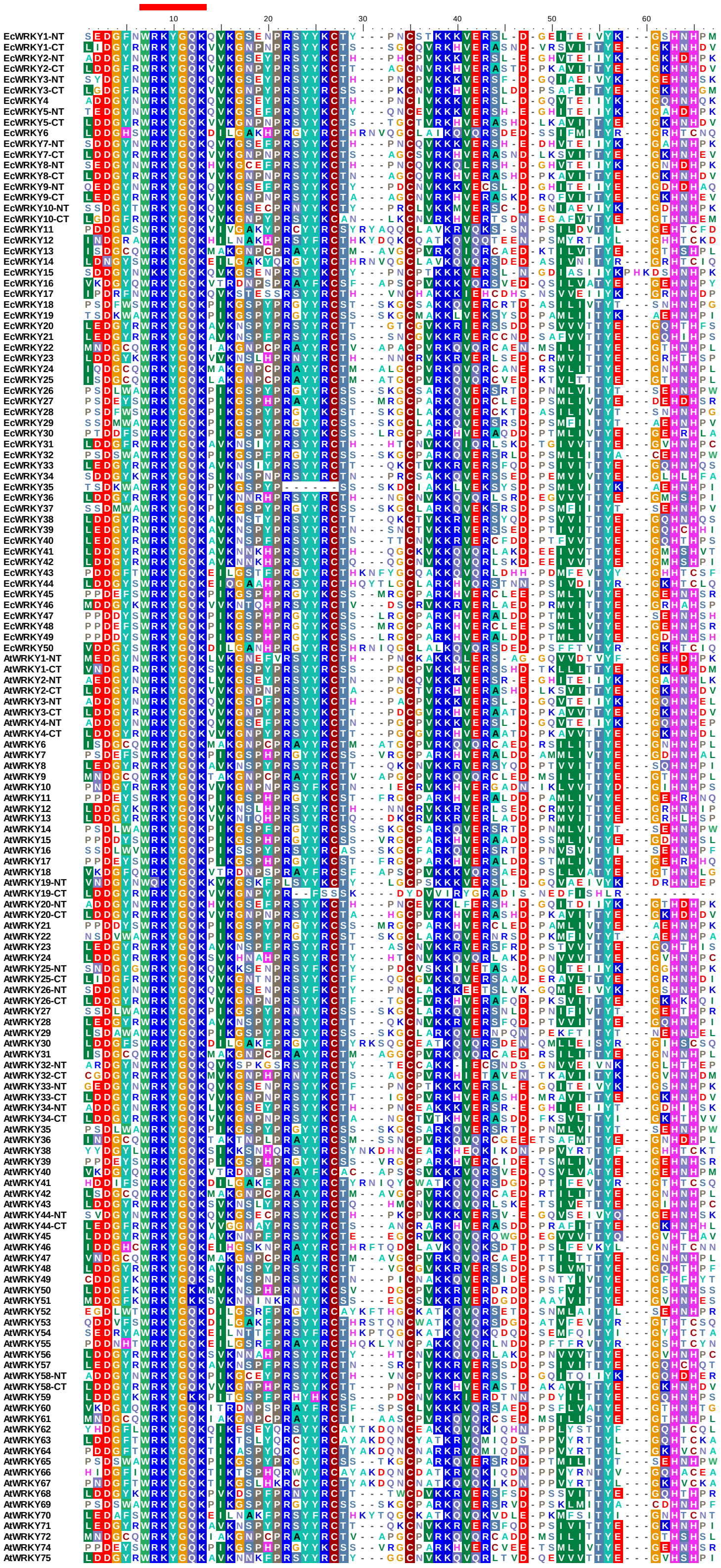

Supplementary Figure 2. Multiple sequence alignment of the WRKY domains of *Eschscholzia californica* and *Arabidopsis thaliana* WRKY transcription factors. Red box indicates the WRKYGQK core sequences.

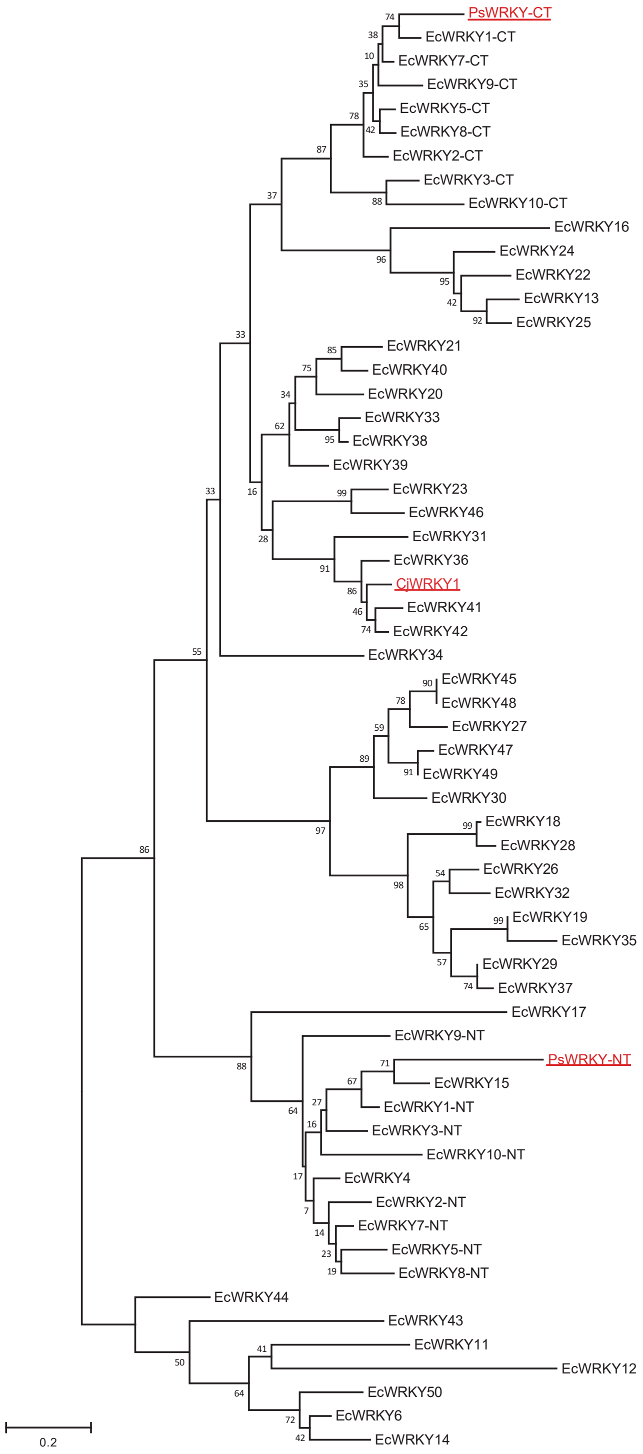

Supplementary Figure 3. Phylogenetic tree of EcWRKY proteins with CjWRKY1 and PsWRKY. An neighbor-joining tree was constructed based on the amino acid sequences of the WRKY domains in 50 EcWRKY proteins, CjWRKY1 (accession no. AB267401), and PsWRKY (accession no. JQ775582) using MEGA 7.0. Bootstrap confidence values from 1,000 replicates are indicated at each branch.

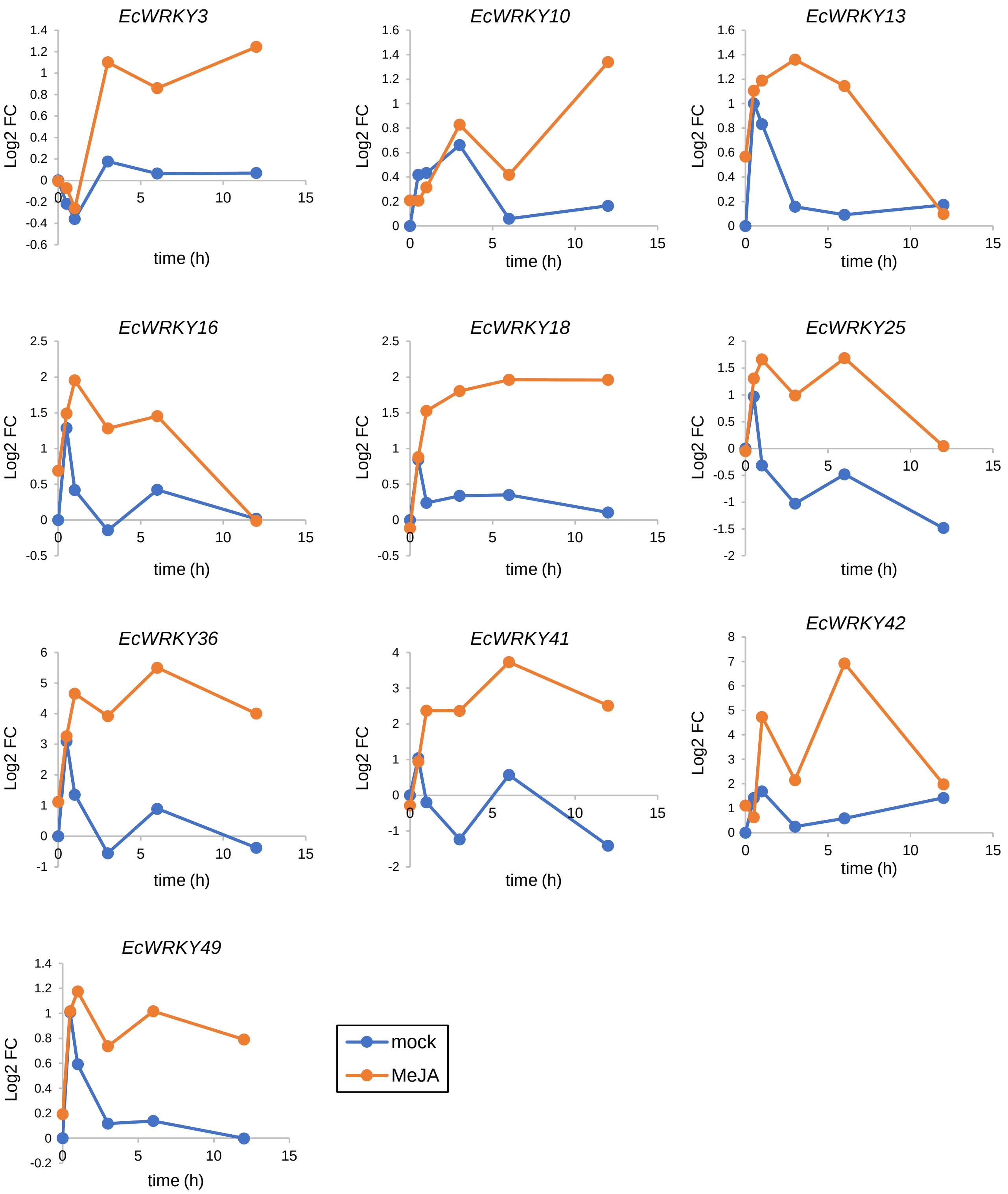

Supplementary Figure 4. Expression patterns of MeJA-induced *EcWRKY* genes. Temporal expression patterns of *EcWRKY* genes upregulated by MeJA are shown with log2 FC values based on the RNA-Seq data.

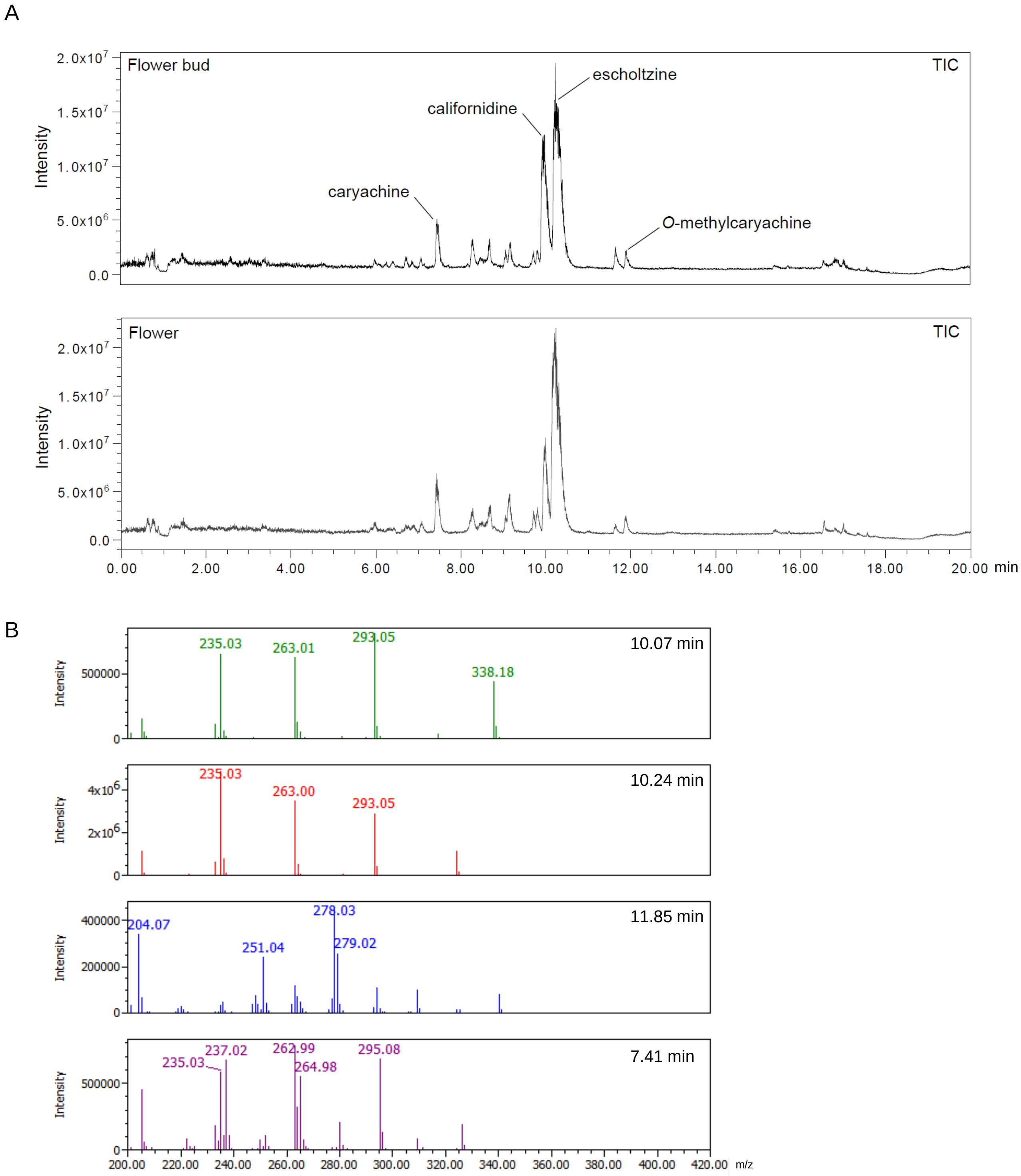

Supplementary Figure 5. Alkaloid profile of floral organs in California poppy. Total ion chromatograms (A) and mass spectrometric fragment patterns (B) are presented. The predicted molecular masses of californidine, escholtzine, caryachine, and *O*-methylcaryachine were m/z 338, $[M+H]^+$, m/z 324, $[M+H]^+$, m/z 326, $[M+H]^+$, and m/z 340 $[M+H]^+$, respectively.

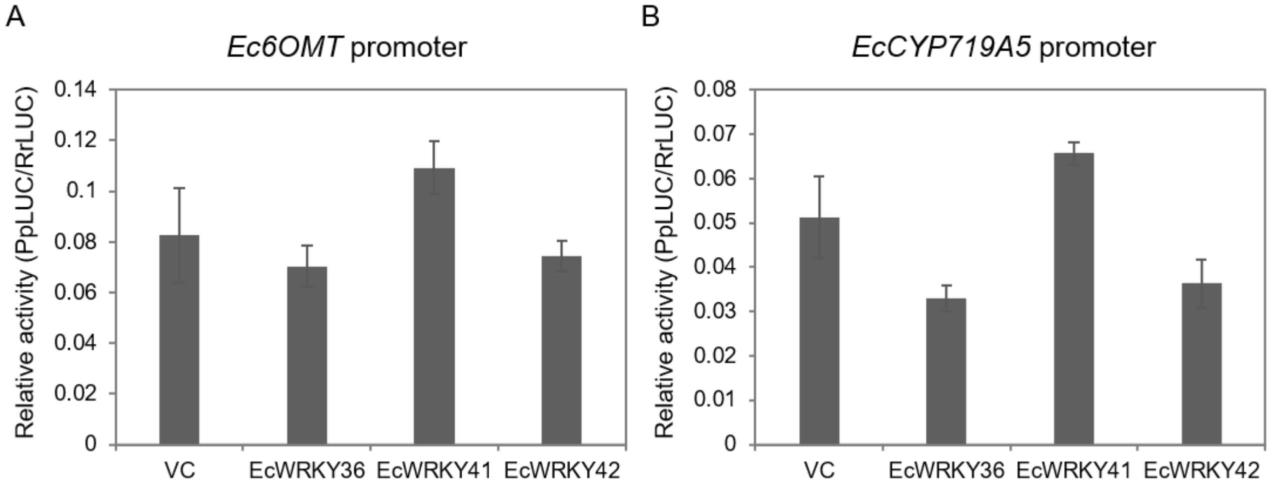

Supplementary Figure 6. Transcriptional activity of subgroup IIc EcWRKY proteins. Transient luciferase reporter assay was performed using the *Ec6OMT* (A) and *EcCYP719A5* (B) promoter::*LUC* reporter constructs. The pBI221 empty vector was also introduced into protoplasts as a vector control (VC). Luciferase activity was determined using a dual-luciferase reporter assay. Error bars indicate the standard deviations calculated from three biological transfections.
